# Serum Neurofilament Light Chain Predicts Stroke Outcome and is a Potential Marker for Treatment Effects of Neural Stem Cell-derived Extracellular Vesicles in a Rat Stroke Model

**DOI:** 10.1007/s12975-026-01445-6

**Published:** 2026-05-08

**Authors:** Megan K. Cannon, Alinde R. Fojtik, Charles M. White, Raymond L. Swetenburg, Steven L. Stice, Sean I. Savitz, Emily W. Baker

**Affiliations:** 1 Aruna Bio Inc, Athens, GA USA; 2 Regenerative Bioscience Center, University of Georgia, Athens, GA USA; 3 Department of Animal and Dairy Science, University of Georgia, Athens, GA USA; 4 Institute for Stroke and Cerebrovascular Disease, UTHealth-Houston, Houston, TX USA

**Keywords:** Ischemic Stroke, Neural stem cell derived extracellular vesicles, Biomarkers, Neurofilament light chain

## Abstract

Acute ischemic stroke (AIS) remains a leading cause of disability worldwide, and effective treatments are urgently needed beyond reperfusion therapy. Translating preclinical success to clinical impact has been hindered by variability in animal models and the lack of translational biomarkers that predict outcomes across species. To overcome these barriers, we developed a robust rat AIS model optimized for consistency and severity, enabling rigorous therapeutic testing. Additionally, we tested a panel of common clinical serum biomarkers to improve translation from rodents to humans. We demonstrated that serum neurofilament light chain (NfL) -a biomarker widely used in clinical stroke studies- strongly correlated with functional outcomes, establishing a translational link that has not been previously reported in rats. Notably, NfL’s predictive capabilities performed as well as infarct volume while outperforming serum biomarkers intercellular adhesion molecule-1 (ICAM-1) and S100 calcium binding protein (S100B). Using this platform, we evaluated the therapeutic impact of neural stem cell-derived extracellular vesicles (NSC EV), a novel biologic therapy poised for clinical trials, on stroke outcome in our rat AIS model. A three-dose regimen of NSC EV over 48 h confirmed our previous studies and produced the best outcomes in stroked animals evidenced by smaller infarct volume, improved neurologic score, and reduced serum NfL. In addition, single-dose and two-dose regimens of NSC EV were both effective at some endpoints. These findings not only validate NfL as a cross-species biomarker but also provide critical dosing insights for NSC EV therapy, accelerating the path from bench to bedside for AIS treatment.

**Supplementary Information:**

The online version contains supplementary material available at 10.1007/s12975-026-01445-6.

## Introduction

While reperfusion therapy is the gold standard intervention for acute ischemic stroke (AIS), less than half will be functionally independent at Day 90 even when complete cerebral reperfusion is achieved [1–3]. Furthermore, reperfusion therapy does not address the complex cascade of cellular and molecular events triggered by ischemia. Additional treatment options are an urgent, global unmet medical need. Neural stem cell-derived extracellular vesicles (NSC EV) are a novel biopharmaceutical product with powerful, multi-mechanistic target biology, and previous studies published by our lab demonstrate that NSC EV reduces infarct size and mitigates the systemic immune response leading to preserved motor function in multiple stroke models [4–6]. Additionally, NSC EV provides unique advantages to cell-based therapies due to its manufacturing scalability, ease of administration, and the ability to cross the blood brain barrier and home to the infarct site [7]. While other biologic therapies including NSC EV show promise in stroke models, it is challenging to translate preclinical results to clinical trials.

Identifying reliable biomarkers that translate from preclinical to clinical studies is a gap that hinders new stroke therapies. Robust and consistent blood-based biomarkers could aid in AIS prognosis and serve as a surrogate biomarker for treatment effect, while being less expensive and more accessible than neuroimaging or cerebrospinal fluid (CSF)-based biomarkers [8]. Establishing a biomarker that is linked, either directly or indirectly, to the investigational drug’s action is critical for clinical trial success, as it enables highly quantitative pharmacological assessment and strengthens the biological rationale for therapeutic efficacy [9]. There have been promising candidates for determining AIS prognosis as well as differentiating between stroke types [10, 11]. Recent clinical studies of anti-inflammatory and neuroprotective investigational drugs for AIS have measured serum levels of pro-inflammatory mediators, such as interleukin-6 (IL-6), interleukin-1 beta (IL-1β), and tumor necrosis factor-alpha (TNFα) [12, 13]. However, no recent trials have successfully met their primary endpoint after Phase 2 despite promising preclinical blood-based biomarker data [12, 13]. This could possibly be due to species differences in post-AIS inflammatory signaling and cell death mechanisms [14]. The temporal, and often transient, dynamic changes in the serum concentration of these proteins in AIS patients, which may vary based on stroke severity and/or type, could also contribute to a lack of meaningful readouts.

The primary objective of this study was to build upon our previous pharmacology studies by identifying a prognostic biomarker that correlates with NSC EV activity and remains informative across relevant therapeutic windows for extended dosing paradigms. A robust rat transient middle cerebral artery occlusion (tMCAO) model presents an opportunity to develop a biomarker that can directly bridge preclinical data to clinical endpoints. In the absence of validated biomarkers for stroke outcome, we selected S100 calcium-binding protein B (S100B), intercellular adhesion molecule-1 (ICAM-1), and neurofilament light chain (NfL) based on our NSC EV pharmacology data as well as studies published by other groups. Previous studies have demonstrated that S100B correlates with infarct size and neurological outcomes in both preclinical rat models and human patients [15–17]. Similarly, elevated ICAM-1 levels have been associated with worse outcomes in AIS patients [18]. In addition, ICAM-1 plays a key role in blood–brain barrier disruption and leukocyte infiltration, supporting its relevance as a candidate biomarker given our data showing that NSC EV treatment mitigates hemorrhagic transformation in a pig stroke model [5]. Finally, NfL has emerged as a promising biomarker for AIS prognosis. NfL is a well-established marker for neuroaxonal injury or degradation and has been studied in many neurological diseases including multiple sclerosis [19], amyotrophic lateral sclerosis (ALS) [20], traumatic brain injury (TBI) [21], and Alzheimer’s disease [22–26]. Clinical studies have shown a strong association between elevated NfL concentration, larger cerebral infarct volume, and poor functional outcomes in AIS patients, demonstrating its potential as a robust serum biomarker for AIS [27–33]. Despite these findings, few preclinical studies have evaluated serum NfL levels after stroke: both studies have been in mice and only one measured NfL levels longitudinally [34, 35].

In this study we addressed this clinical translation roadblock by first determining that an occlusion time of 150 min was optimal for tMCAO, resulting in consistently large infarcts comprising both cortical and subcortical regions, which corresponded with significant functional impairment. Next, we found that serum levels of NfL, but not S100B or ICAM-1, significantly correlated with AIS severity, while NfL performed as well as lesion size as a predictor of good functional outcomes. Lastly, we demonstrated the therapeutic potential of NSC EV at 1-, 2-, and 3-doses with improvements in neurologic deficit, infarct volume, and serum NfL. These results demonstrate that the pharmacological effect of a novel investigational biologic product, NSC EV, can be robustly quantitated by changes in serum NfL levels in a preclinical tMCAO model. Furthermore, these results provide proof-of-concept that serum NfL can be utilized as a biomarker to optimize dose paradigms in the preclinical setting to be translated to the clinic, offering a new strategy to rigorously assess promising investigational drugs and bring new treatment options to stroke patients.

## Methods

### Experimental Groups

This study included two experiments: (1) development of the surgical model by comparing the effect of occlusion times on body weight, neurologic deficit score, and cerebral infarct volume, and (2) evaluating the effect of NSC EV on body weight, neurologic deficit score, cerebral infarct volume, and serum levels of NfL, S100B, and ICAM-1. For Experiment 1, rats were placed into the following groups: (1) 90 min occlusion (*n* = 16), (2) 120 min occlusion (*n* = 16) and (3) 150 min occlusion (*n* = 17). For Experiment 2, rats were placed in the following groups (*n* = 6–20): (1) untreated, (2) one NSC EV dose, (3) two NSC EV doses, or (4) three NSC EV doses. The three-dose regimen was designed to provide therapeutic coverage during the acute post-stroke period. NSC EV was administered at 4 h post-reperfusion to prioritize early intervention, consistent with prior pharmacology studies. Additional doses at 24 and 48 h post-stroke were included to maintain systemic exposure during the acute phase, based on previously biodistribution data demonstrating clearance of NSC EV from the infarct region within 24 h [6]. Since the pharmacology of the 3-dose regimen was previously established [5, 6], the 1-dose and 2-dose regimens were added to determine whether the therapeutic effects of the 3-dose regimen are dose-dependent or time-dependent.

### Animals

All experimental protocols performed in this study were reviewed, approved, and complied with the guidelines established by the University of Georgia Institutional Animal Care and Use Committee. All studies utilized adult male Wistar rats (7 weeks old, 220–270 g at the time of surgery) to minimize biological variability. Rats were obtained from Charles River Laboratories (Wilmington, MA) and allowed to acclimate to the facility for one week prior to surgery. Rats were pair-housed prior to surgery, and individually housed after surgery, on a 12 h light/dark cycle and given food and water ad libitum.

### Transient Middle Cerebral Artery Occlusion (tMCAO)

Transient focal ischemia was induced by occlusion of the right middle cerebral artery (MCA) using the silicon-coated suture insertion method [36, 37]. For anesthesia induction, an induction chamber was filled with 5% isoflurane with oxygen. 1–2% isoflurane with oxygen was used for anesthesia maintenance. All rats were subcutaneously administered Ropivacaine (3 mg/kg) for local analgesia to the incision site and 0.65 mg/kg sustained release Buprenorphine (Ethiqa XR^®^) for systemic analgesia. Throughout the procedure, body temperature was maintained at 37 °C using a heating pad. A midline neck incision was made, and the right internal, external, and common carotid arteries (ICA, ECA, CCA) were exposed. After cutting a small hole in the ECA, a silicon coated monofilament suture (Doccol Corporation 403756) was advanced through the ICA lumen to the MCA, until resistance was felt. The surgical incision was closed and animals recovered in their home cage under a heat lamp. Proper monofilament placement, resulting in cerebral ischemia, was confirmed by assessing abnormalities in front paw flexion and circling behavior prior to reperfusion. Animals were excluded if they displayed normal behavior (neurologic score of 8) during these tests. After the MCA was occluded for 90, 120, or 150 min for Experiment 1, and 150 min for Experiment 2, rats were re-anesthetized, and the filament was withdrawn from the ICA to restore cerebral perfusion. Animals were excluded from the study if they scored normal (neurologic score of 8) approximately 4–5 h after stroke (Day 0). Of all animals enrolled in the study, only one animal was excluded due to this criterion being met. Rats were weighed prior to surgery and daily on Days 0–3. A humane endpoint of 30% loss of pre-stroke body weight was prospectively set in the study protocol; however, no animals met this endpoint prior to the scheduled Day 3 endpoint.

### NSC EV Formulation and Intravenous Administration

Cryopreserved vials of the neural stem cell (NSCs) were thawed and expanded in bulk in suspension bioreactors until the target volume of cell culture conditioned media containing EVs was generated. Next, the conditioned media underwent a series of tangential flow filtration (TFF) and chromatography steps to separate and concentrate the EVs. The EVs underwent Nanoparticle Tracking Analysis (NanoSight, Malvern Panalytical), single-particle interferometric reflectance imaging (Unchained Labs) and protein quantification (ThermoFisher) (Supp. Figure 1). The final NSC EV preparation was made by diluting the EVs with formulation buffer to the target concentration. The bulk NSC EV preparation was filled into sterile polypropylene vials as individual doses (2.7e11 particles per kilogram body weight) and stored at -20 °C. On the day of dosing, NSC EV vials were thawed at 4 °C. Rats were anesthetized with isoflurane and administered NSC EV (460 µl) intravenously via the lateral tail vein.

### Neurologic Deficit Assessment

All rats were assessed for neurological deficit using a modified version of the Bederson test [38] at 4 h after stroke reperfusion (i.e., Day 0) as well as Days 1 and 3 post-stroke. Front paw flexion, back paw flexion, circling behavior, and resistance to push were scored on a scale of 0–2, where 0 indicates severe deficit, 1 indicates moderate deficit, and 2 is normal. A total score of 0–8 was then calculated. For receiver operating characteristics (ROC) curves, a good outcome was defined as a neurologic score of 6 or greater on Day 3, while a poor outcome was defined as a neurologic score of 5 or lower. Animals with poor outcomes displayed significant impairment that disrupted quality of life such as trouble accessing and consuming standard rodent chow. Animals with a good outcome exhibited minimal impairment of everyday functioning.

### Serum Processing and Measurement of Serum Biomarkers

Blood samples were collected from the ventral tail artery of all animals on Days 1, 2, and 3 post-stroke. Samples were placed into serum separator tubes, kept at room temperature for 30 min to allow for coagulation then centrifuged at 4000 x g for 10 min to separate serum. NfL (UMAN Diagnostics), ICAM-1 (Invitrogen), and S100B (Invitrogen) proteins were quantified according to the manufacturer’s instructions. All plates were analyzed using the SpectraMax id5 Multimode Microplate Reader (Molecular Devices).

### Evaluation of Infarct Volume

All animals were euthanized on Day 3 by transcardial perfusion with phosphate buffer saline. The whole brain was extracted immediately then sliced coronally into 6 consecutive 2 mm sections using a rat brain slicer matrix (Zivic Instruments). Animals displaying cerebral hemorrhage were excluded from the study. The extent of cerebral infarct was evaluated by placing sections in 2% 2,3,5-Triphenyltetrazolium chloride (TTC, Sigma-Aldrich) and incubating for 10 min at 37 °C. The area of infarct (i.e., colorless tissue), ipsilateral hemisphere, and contralateral hemisphere was measured for each section using Fiji ImageJ 2.14 (NIH, Bethesda, USA). The areas for each section were multiplied by slice thickness (2 mm) then summed into a total volume. Total hemispheric lesion volume with edema correction (%HLVe) was calculated as a percentage using the following formula [39]:

$$\begin{aligned}&\\&\%HLVe=&\\&\frac{Contralateral\:Volume-(Ipsilateral\:Volume-Lesion\:Volume)}{Contralateral\:Volume}\:\times\:100\end{aligned}$$

### Statistical Analyses

Line graphs were illustrated with mean ± standard error of the mean (SEM). Violin plots were illustrated with median and quartiles. All data were analyzed using GraphPad Prism 10.4.2 and/or JMP 18/19. Statistical analysis of neurologic deficit and body weight was performed using two-way analysis of variance (ANOVA), or a Mixed-Effects Model in cases of unbalanced datasets, followed by post-hoc Tukey’s Pair-Wise comparisons. Pearson correlation and ROC curves were generated and analyzed with JMP. For ROC curves, a good outcome was defined as a neurological deficit score of 6 or greater. Model performance was compared using the Model Comparison platform in JMP 19. Differences between AUCs were evaluated using a Likelihood Ratio Chi-Square test, with a significance level of α = 0.05. Figures were prepared using GraphPad Prism 10.4.1 and Adobe Photoshop 2022.

## Results

### Increasing Occlusion Time to 150-minutes Results in Higher Stroke-induced Weight Loss and Infarct Volume, as well as Decreased Incidence of Subcortical Infarcts

It is critical for laboratories to optimize their model and endpoints prior to treatment studies so that it most closely mimics the intended clinical population. We have previously shown that NSC EV effects are positively correlated with stroke severity, with the most robust treatment response observed in animals with poorer prognosis [4]. Therefore, our goal was to rigorously optimize a preclinical tMCAO model with a highly reproducible and severe neural injury. The model optimization study design is summarized in Supp. Figure 2a. Neurologic deficit was measured before stroke and on Day 0, 1, and 3 post-stroke using a modified version of the Bederson test [40]. Neurologic deficit was represented as a composite score between 0 and 8, where 8 signified no deficit. Neurologic scores for the 90-, 120-, and 150-minute groups were significantly lower than pre-stroke on Day 0, Day 1, and Day 3 (*p* < 0.05, Supp. Figure 2b, Table 1). The 120-minute group had significantly lower neurologic scores on Day 1 compared to Day 0 (*p* < 0.05, Supp. Figure 2b, Table 1). These results confirm stroke induction for all occlusion times which is evident by significantly decreased neurologic scores persisting through Day 3.

**Table 1:** Neurologic deficit score between occlusion times

	Pre-stroke	Day 0	Day 1	Day 3
90-minutes	8.0 ± 0.0	5.6 ± 0.2*	5.0 ± 0.6*	5.4 ± 0.1*
120-minutes	8.0 ± 0.0	5.4 ± 0.2*	4.9 ± 0.2*^#^	5.4 ± 0.2*
150-minutes	8.0 ± 0.0	5.2 ± 0.3*	4.9 ± 0.2*	4.9 ± 0.2*

Data is expressed as a composite score between 0–8. Mean ± SEM. * indicates significant difference from Pre-Stroke and ^#^ indicates significant difference from Day 0

Body weight, represented as a percentage of pre-stroke, was measured for 90-, 120-, and 150-minute groups before stroke and on Day 0, 1, 2, and 3 post-stroke. Body weights in the 120- and 150-minute groups were significantly lower than pre-stroke on Day 0, Day 1, Day 2, and Day 3 (*p* < 0.05, Supp. Figure 2c, Table 2). In the 90-minute group, there was no difference in body weight between pre-stroke and Day 0, Day 1, or Day 3, but body weight was significantly lower on Day 2 compared to pre-stroke (*p* < 0.05, Supp. Figure 2c, Table 2). In the 120-minute group, body weight on Day 1 was significantly lower compared to Day 0 (*p* < 0.05, Supp. Figure 2c, Table 2). For the 150-minute group, body weights on Day 1, Day 2, and Day 3 were significantly lower than Day 0 (*p* < 0.05, Supp. Figure 2c, Table 2). Furthermore, in the 150-minute group, body weights on Day 2 and Day 3 were significantly lower than on Day 1 (*p* < 0.05, Supp. Figure 2c, Table 2). Taken together, these results show that occlusion time is associated with the degree of stroke-induced weight loss, with the 150-minute group demonstrating greater weight loss over Days 0–3 compared to the 90-minute and 120-minute groups.

**Table 2:** Body weight between occlusion times

	Pre-stroke	Day 0	Day 1	Day 2	Day 3
90-minutes	100.0 ± 0.0	95.9 ± 1.4	93.2 ± 2.1	89.6 ± 2.7*	87.1 ± 3.6
120-minutes	100.0 ± 0.0	93.7 ± 0.5*	91.1 ± 1.3*^#^	87.6 ± 2.3*	86.5 ± 2.7*
150-minutes	100.0 ± 0.0	93.8 ± 0.6*	88.5 ± 0.9*^#^	82.0 ± 2.0*^#&^	79.5 ± 2.8*^#&^

Data is expressed as a percentage of Pre-stroke body weight. Mean ± SEM. * indicates significant difference from Pre-Stroke, ^#^ indicates significant difference from Day 0 and ^&^ indicates significant difference from Day 1

Cerebral infarct volume, represented as a percentage of edema corrected hemispheric lesion volume (%HLVe), was measured on Day 3 through TTC staining. Animals in the 150-minute group had significantly higher infarct volume than both 90- and 120-minute groups (*p* < 0.05, Supp. Figure 2d, Table 3). Furthermore, we categorized the data by infarct type, i.e., whether the cerebral infarct spanned both cortical and subcortical regions or subcortical regions only, and found that the 150-minute group had higher incidence of infarcts spanning both the cortical and subcortical regions when compared to 90- and 120-minute groups (88% vs. 69% and 62%, Supp. Figure 2e-f).

**Table 3:** Infarct volume between occlusion times

	Day 3
90-minutes	37.3 ± 4.3^a^
120-minutes	33.0 ± 3.7^a^
150-minutes	50.4 ± 2.8^b^

Data is expressed as total hemispheric lesion volume with edema correction (%HLVe). Mean ± SEM. Different letters indicate significant differences between groups

Overall, these data demonstrate that a 150-minute occlusion time leads to greater infarct volume and reduced incidence of subcortical infarcts compared to the 90- and 120-minute occlusion times. Since NSC EV has been shown to confer greater benefit in severe strokes [41], these results identify an occlusion time of 150 min as the optimal model for subsequent treatment studies.

### Elevated Concentration of Serum NfL is Associated with Increased Stroke Deficit and Predictive of Good Neurologic Outcomes

**Fig. 1:**
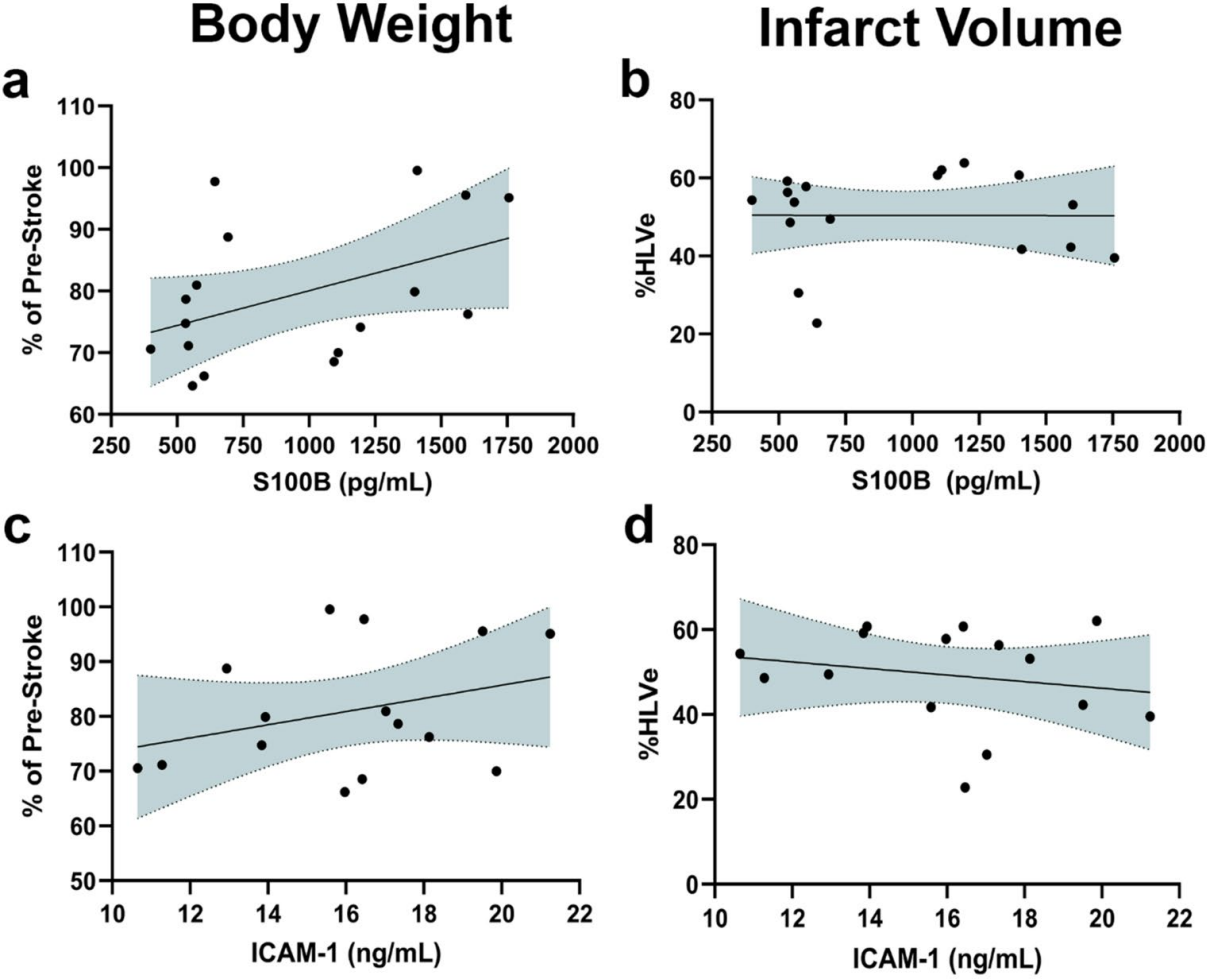
Neither serum S100B nor ICAM-1 are strongly correlated with body weight and infarct volume. Correlation of Day 3 S100B (*n* = 17) with (**a**) body weight and (**b**) infarct volume. Correlation of Day 3 ICAM-1 (*n* = 15) with (**c**) body weight and (**d**) infarct volume

Univariate correlation analyses were performed to examine the relationship between three serum biomarker concentrations (S100B, ICAM-1, and NfL) with body weight and infarct volume. There was no significant correlation between body weight and S100B (*r* = 0.45, *p* = 0.07) or ICAM-1 (*r* = 0.32, *p* = 0.24) serum concentration on Day 3 (Fig. 1a, c; Table 4). There was also no significant correlation between infarct volume and serum concentration of S100B (*r* = -0.003, *p* = 0.99) and ICAM-1 (*r* = -0.20, *p* = 0.47) on Day 3 (Fig. 1b, d; Table 4). These results suggest that serum S100B and ICAM-1 are not robust biomarkers for this AIS model during the window of interest, therefore they were not investigated in subsequent studies.

**Table 4:** Correlations between Day 3 body weight, HLVe, S100B, ICAM-1, and NfL

Serum marker	Comparator	*r* value	Lower 95% CI	Upper 95% CI	*p* value
S100B	%HLVe	0.00	-0.4828	0.4785	0.99
S100B	Body Weight	0.45	-0.04068	0.7645	0.07
ICAM-1	%HLVe	-0.20	-0.6472	0.3462	0.47
ICAM-1	Body Weight	0.32	-0.2294	0.7153	0.24
Day 1 NfL	%HLVe	0.30	-0.003762	0.543	0.0537
Day 1 NfL	Body Weight	-0.38	-0.5876	-0.06167	0.0143
Day 2 NfL	%HLVe	0.78	0.6054	0.8774	< 0.0001
Day 2 NfL	Body Weight	-0.65	-0.8056	-0.4234	< 0.0001
Day 3 NfL	%HLVe	0.76	0.5981	0.8586	< 0.0001
Day 3 NfL	Body Weight	-0.80	-0.8813	-0.6548	< 0.0001

**Fig. 2:**
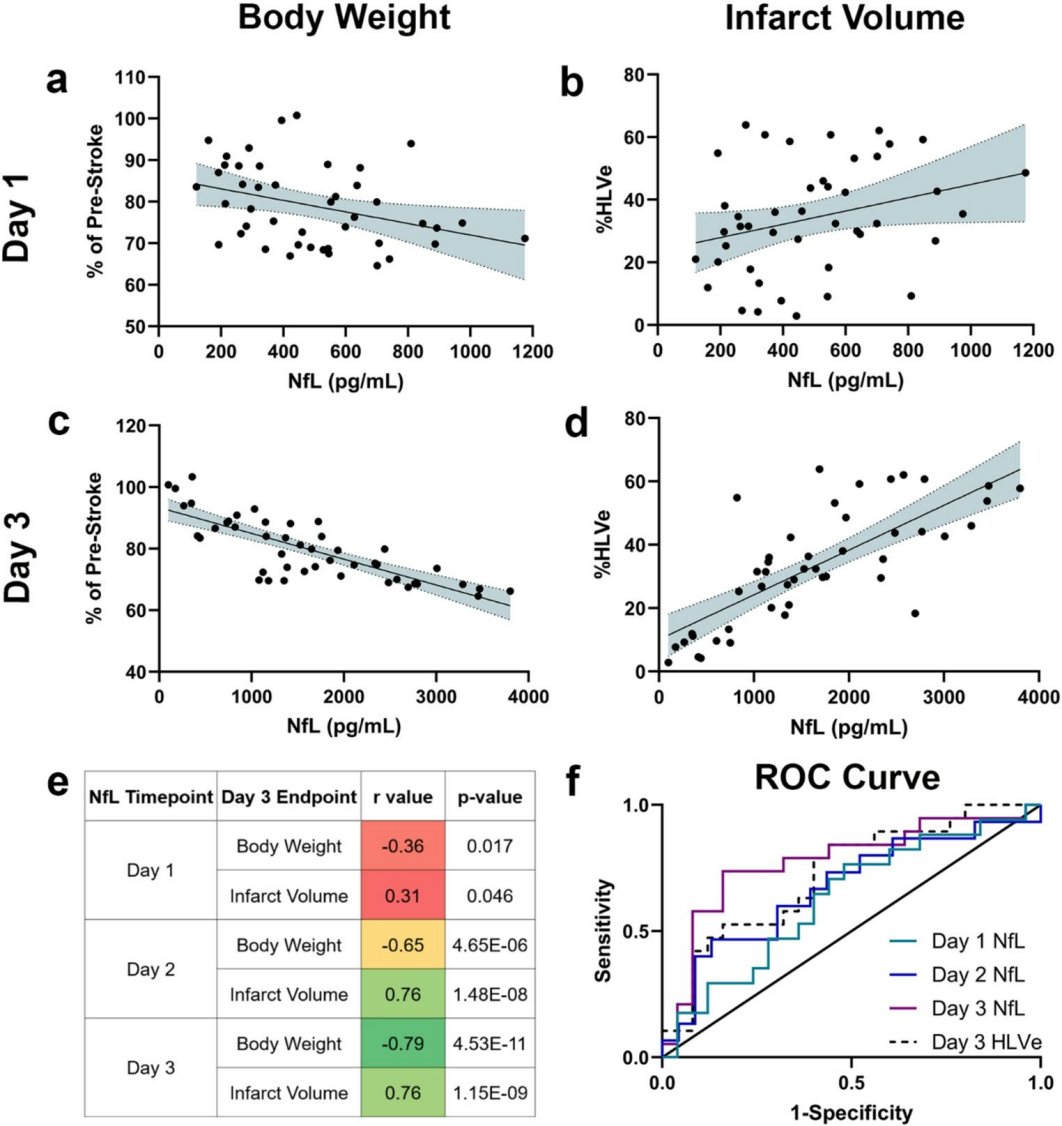
NfL correlates with body weight loss and infarct volume and is predictive of good functional outcomes. Correlation of NfL with (**a**) body weight or (**b**) infarct volume at Day 1 (*n* = 44). Correlation of NfL with (**c**) body weight or (**d**) infarct volume at Day 3 (*n* = 46). (**e**) Table showing r value and p-value between NfL and body weight or infarct volume across the three time points. (**f**) Receiver operating characteristic curve for good functional outcomes of Day 1 NfL (green line), Day 2 NfL (blue line), Day 3 NfL (purple line) and lesion size (dashed line)

There was a significant correlation between serum NfL concentration and body weight (*r* = -0.36, *p* < 0.05) as well as infarct volume (*r* = 0.31, p *<* 0.05) on Day 1 (Fig. 2a-b, e; Table 4). The strength of the correlation between NfL with body weight (*r* = -0.65, *p* < 0.01) and infarct volume (*r* = 0.76, *p* < 0.01) increased considerably on Day 2 (Fig. 2e; Supp. Figure 3, Table 4). The correlation between NfL and body weight (*r* = -0.79, *p* < 0.01) and infarct volume (*r* = 0.76, *p* < 0.01) was strongest on Day 3 (Fig. 2c-e; Table 4). These results show that the relationship between serum NfL concentration and worsening body weight and infarct volume are highly correlated and strengthens over time.

To investigate whether serum NfL level at Day 1, 2 or 3, or infarct volume on Day 3 could discriminate between good and poor functional outcomes, we performed ROC analysis. A good outcome was defined as a neurologic score of 6 or greater on Day 3, while a bad outcome was defined as a neurologic score of 5 or lower. Neither Day 1 nor Day 2 NfL ROC analyses could delineate functional outcomes (Day 1: *p* = 0.164, AUC = 0.628 and Day 2: *p* = 0.086, AUC = 0.672, Fig. 2f; Table 5). However, Day 3 NfL successfully differentiated between good and poor functional outcomes (*p* = 0.002, AUC = 0.779, Fig. 2f; Table 5). Notably, Day 3 NfL level performed as well as infarct volume (*p* = 0.0051, AUC = 0.726, Fig. 2f; Table 5). Infarct volume had a slightly higher sensitivity than Day 3 NfL at its respective cutoff point (84.2% vs. 73.7%) but had a much lower specificity (84.0% vs. 56.0%). As Day 3 NfL and HLVe were the only significant models, we compared each model pairwise. When comparing models, Day 1 NfL was not significantly different than any of the other models (Table 6). Day 2 NfL was significantly different than both Day 3 NfL (*p* < 0.0001) and infarct volume (*p* = 0.0445), while Day 3 NfL and infarct volume were not significant (*p* = 0.342, Table 6). Thus, the results indicate that NfL is likely too noisy to be predictive at earlier time points, but as the signal emerges on Day 3, it functions as well as infarct volume for predicting outcomes. Taken together, these data suggest that serum NfL is a robust biomarker for AIS severity and can serve as an indicator of functional outcome in both preclinical and clinical settings.

**Table 5:** ROC curve analysis on NfL and HLVe

	AUC	Lower 95% CI	Upper 95% CI	Prob> ChiSq
Day 1 NfL	0.6282	0.4438	0.7816	0.1636
Day 2 NfL	0.6725	0.4695	0.8265	0.0855
Day 3 NfL	0.7789	0.597	0.8934	0.0017
%HLVe	0.7263	0.5517	0.8513	0.0051

**Table 6:** ROC model comparisons

Level 1	Level 2	Prob> ChiSq
Day 1 NfL	Day 2 NfL	0.6692
Day 1 NfL	Day 3 NfL	0.0946
Day 1 NfL	%HLVe	0.3454
Day 2 NfL	Day 3 NfL	< 0.0001
Day 2 NfL	%HLVe	0.0445
Day 3 NfL	%HLVe	0.342

### NSC EV Treatment Significantly Improves Stroke-induced Weight Loss, Neurologic Deficit, Infarct Volume, and Elevated NfL Concentration

**Fig. 3:**
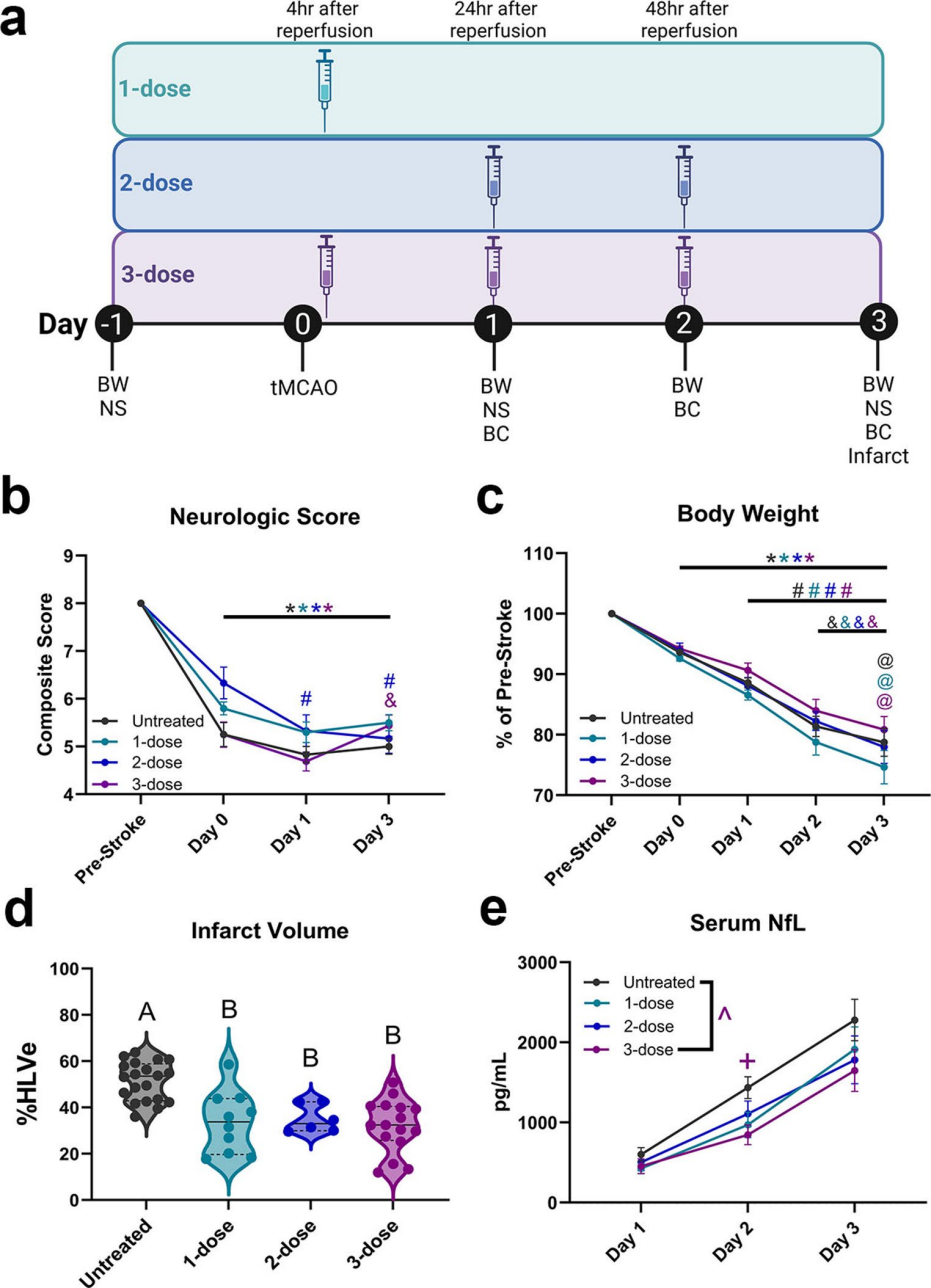
NSC EV treatment significantly improves stroke-induced weight loss, neurologic deficit, infarct volume, and elevated NfL concentration. (**a**) Experimental outline showing treatment schedules and pharmacological timepoints relative to tMCAO surgery. (**b**) Neurologic score collected pre-stroke and Day 0, 1, and 3. (**c**) Body weight data collected pre-stroke and Day 0, 1, 2, and 3. (**d**) Quantification of cerebral infarct volume on Day 3 for all treatment groups. (**e**) Quantification of NfL concentration for all groups on Day 1, 2, and 3. *n* = 20 for the untreated group; *n* = 10 for the 1-dose group; *n* = 6 for the 2-dose group; *n* = 16 for the 3-dose group. Data expressed as Mean ± SEM. * indicates *p* < 0.05 compared to pre-stroke; # indicates *p* < 0.05 compared to Day 0; & indicates *p* < 0.05 compared to Day 1; @ indicates *p* < 0.05 compared to Day 2. For panel **a**, ***BW***: body weight, ***NS***: neurologic score, and ***BC***: blood collection. For panel **c**, levels not connected by the same letter are significantly (*p* < 0.05) different. For panel **e**, ^ indicates significant (*p* < 0.05) main effect between untreated vs. 3-dose; + indicates *p* < 0.05 between untreated vs. 3-dose

We utilized the 150-minute tMCAO model to directly compare the therapeutic effect of three NSC EV dose regimens on stroke outcomes through body weight loss, neurologic score, infarct volume, and serum NfL levels. The dose regimens are outlined in Fig. 3a. Neurologic scores for the untreated, 1-dose, 2-dose, and 3-dose groups on Day 0, Day 1, and Day 3 were significantly lower compared to pre-stroke, which confirms stroke induction (*p* < 0.05, Fig. 3a; Table 7). Neurologic scores for the 2-dose group on Day 1 and Day 3 were significantly lower compared to Day 0 (*p* < 0.05, Fig. 3a; Table 7). The 3-dose group demonstrated a significantly higher neurologic score on Day 3 compared to Day 1 (*p* < 0.05, Fig. 3a; Table 7). This data suggests that the 3-dose NSC EV regimen led to neurologic improvement between Days 1 and 3.

**Table 7:** Neurologic deficit score between treatment groups

	Pre-stroke	Day 0	Day 1	Day 3
Untreated	8.0 ± 0.0	5.3 ± 0.3*	4.8 ± 0.2*	5.0 ± 0.2*
1-dose	8.0 ± 0.0	5.8 ± 0.1*	5.3 ± 0.2*	5.5 ± 0.2*
2-dose	8.0 ± 0.0	6.3 ± 0.3*	5.3 ± 0.3*^#^	5.2 ± 0.3*^#^
3-dose	8.0 ± 0.0	5.3 ± 0.3*	4.7 ± 0.2*	5.4 ± 0.2*^&^

Data is expressed as a composite score between 0–8. Mean ± SEM. * indicates significant difference from Pre-Stroke, ^#^ indicates significant difference from Day 0 and ^&^ indicates significant difference from Day 1

Body weights for the untreated, 1-dose, 2-dose, and 3-dose groups were significantly lower on Day 0, Day 1, Day 2, and Day 3 compared to pre-stroke (*p* < 0.05, Fig. 3b; Table 8). In all four groups, body weight was significantly lower on Day 1, Day 2, and Day 3 compared to Day 0 (*p* < 0.05, Fig. 3b; Table 8). In all four groups, body weight was significantly reduced on Day 2 and Day 3 compared to Day 1 (*p* < 0.05, Fig. 3b; Table 8). The untreated, 1-dose, and 3-dose groups had significantly lower body weight on Day 3 compared to Day 2 (*p* < 0.05, Fig. 3b; Table 8). Together, these data demonstrate that none of the three NSC EV dose regimens mitigated stroke-induced weight loss during the first three days post-stroke.

**Table 8:** Body weight between treatment groups

	Pre-stroke	Day 0	Day 1	Day 2	Day 3
Untreated	100.0 ± 0.0	93.6 ± 0.5*^#&@^	88.6 ± 0.8*^#&@^	81.4 ± 1.7*^#&@^	78.7 ± 2.3*^#&@^
1-dose	100.0 ± 0.0	92.6 ± 0.4*^#&@^	86.6 ± 0.8*^#&@^	78.8 ± 2.1*^#&@^	74.6 ± 2.8*^#&@^
2-dose	100.0 ± 0.0	93.8 ± 1.3*^#&@^	88.1 ± 1.4*^#&@^	82.3 ± 1.5*^#&@^	78.0 ± 2.7*^#&^
3-dose	100.0 ± 0.0	94.2 ± 0.5*^#&@^	90.6 ± 1.2*^#&@^	84.0 ± 1.9*^#&@^	80.9 ± 2.2*^#&@^

Data is expressed as a percentage of pre-stroke body weight. Mean ± SEM. * indicates significant difference from Pre-Stroke, ^#^ indicates significant difference from Day 0, ^&^ indicates significant difference from Day 1, ^@^ indicates significant difference from Day 2

Cerebral infarct volume was measured on Day 3. Infarct volume was significantly lower in the 1-dose, 2-dose, and 3-dose groups compared to untreated animals (*p* < 0.05, Fig. 3c; Table 9; Supp. Figure 4). These results indicate that all three dose regimens significantly reduced cerebral infarct volume by Day 3.

**Table 9:** Infarct volume between treatment groups

	Day 3
Untreated	51.4 ± 1.9^a^
1-dose	33.6 ± 4.2^b^
2-dose	35.1 ± 2.4^b^
3-dose	32.0 ± 2.8^b^

Data is expressed as total hemispheric lesion volume with edema correction (%HLVe). Mean ± SEM. Different letters indicate significant differences between groups

Serum NfL concentration was measured on Days 1, 2, and 3. The 3-dose group had significantly lower levels of serum NfL compared to the untreated group on Day 2 (*p* < 0.05, Fig. 3d; Table 10). There was also a significant main treatment effect (*p* < 0.05), where the 3-dose animals had significantly lower serum NfL than the untreated group across all time points (Fig. 3d; Table 10), suggesting that only the full, 3-dose regimen could reduce NfL levels over time.

**Table 10:** Serum NfL concentration between treatment groups

	Day 1	Day 2	Day 3
Untreated	602.3 ± 84.8	1435.5 ± 135.8	2279.3 ± 258.6
1-dose	425.2 ± 65.2	970.7 ± 159.0	1913.8 ± 279.0
2-dose	502.8 ± 102.4	1108.6 ± 159.0	1782.3 ± 298.6
3-dose^	454.6 ± 90.7	846.5 ± 121.6^+^	1649.1 ± 260.1

Data is expressed as pg/mL. Mean ± SEM. ^ indicates a main effect significant difference from Untreated. ^+^ indicates significant difference from Untreated at Day 2

## Discussion

Novel treatments to aid in stroke recovery after reperfusion are an urgent medical need, however there are barriers that impede the translation of strong preclinical data to positive clinical trial outcomes. The main objective of our study was to identify a prognostic serum biomarker in our rat tMCAO that was responsive to NSC EV treatment effects at clinically actionable time points and could be carried forward to future clinical studies. Two recent clinical studies of anti-inflammatory and neuroprotective investigational products for AIS, ApTOLL and Multistem, measured serum levels of pro-inflammatory mediators as potential biomarkers. ApTOLL was shown to significantly reduce plasma levels of IL-12p70, interferon-gamma (IFNγ), and IL-6 at 24 h after permanent MCAO in mice [42]. However, these results did not translate to significant biomarker changes in AIS patients during their Phase 1b/2a clinical study (APRIL) [12]. There are a couple of possible explanations for this lack of translation. First, it is well-documented that pro-inflammatory cytokines are only transiently elevated in serum after stroke, and preclinical rodent models may demonstrate a more rapid temporal pattern compared to human patients [14]. Therefore, it may be difficult to measure treatment effects at the cytokine’s peak when only a few time points are collected. Second, while mice underwent permanent occlusion, participants in the APRIL trial underwent endovascular thrombectomy (EVT). This key difference in reperfusion status affects both the magnitude and the timing of the inflammatory cytokine response after stroke [14]. Studies in a rat tMCAO model demonstrated that Multistem significantly reduced serum levels of IL-6 and IL-1β four days after intravenous infusion [43]. These findings successfully translated to the MASTERS trial, in which investigators showed that IL-6, IL-1β, and TNFα were significantly reduced on Day 7, unveiling some of the first data showing that an anti-inflammatory product modulates immune responses after AIS in human patients [13]. A potential advantage to the Multistem program, compared to ApTOLL, is that both the rat model and AIS patients were transiently occluded, better harmonizing stroke pathophysiology between the preclinical model and the clinical population. Nonetheless, the lack of progress in developing a blood-based inflammatory cytokine biomarker has likely discouraged its evaluation in other recent AIS trials including Nelonemdaz and ARG-007 [44, 45].

We first evaluated S100B and ICAM-1 as potential serum-based biomarkers to quantitate NSC EV treatment effects in a rat tMCAO model. A previous study in EVT patients showed that serum concentration of S100B correlated with infarct size, and median S100B concentrations were lower on Day 2 in patients with a favorable outcome compared to an unfavorable outcome [15]. Preclinical studies have shown that serum S100B is positively correlated with brain edema, hemorrhage, infarct volume, and neurological outcome in rat tMCAO models [16, 46]. Likewise, serum levels of ICAM-1 have been associated with early neurological deterioration and worse outcome in AIS patients [18, 47]. However, in clinical studies S100B has been shown to peak in the first 24–48 h followed by a rapid decrease [48], and it loses much of its prognostic value by Day 3 [17]. ICAM-1, similarly, peaks around 24 h [49, 50]. Thus, although S100B and ICAM-1 may be well-suited as early post-stroke biomarkers, their early peaks likely explain their limited utility within the Day 3 assessment window of this study.

Given this temporal limitation, we next evaluated neurofilament light chain (NfL), a biomarker with well-described persistence in circulation and emerging relevance for sub-acute stroke outcome assessment. NfL is a neuronal cytoskeleton protein and has been studied as a biomarker for severity at hospital admission and functional prognosis in AIS patients [32, 51]. Plasma levels of NfL are significantly elevated in AIS patients compared to healthy controls up to 6 months post-stroke, with Day 7 levels being the best predictor of moderate-to-high stroke (NIHSS ≥ 5) [52]. However, despite the evidence indicating its utility in clinical AIS, serum NfL in preclinical stroke models remains largely understudied. Plasma NfL was elevated at 24 h through 7 weeks post-stroke in a mouse permanent distal MCAO with hypoxia model, while plasma NfL was elevated on Day 30 in a mouse tMCAO model [34, 35]. Importantly, no prior longitudinal NfL studies have been conducted in a rat stroke model, a species that is widely utilized for AIS drug development. Here, serum NfL correlated with both infarct volume and body weight loss and was predictive of functional outcomes. Our longitudinal results align with a recent report indicating that plasma NfL was positively correlated with infarct volume and independently predicted Day 90 Modified Rankin Score (mRS) more effectively than infarct volume in AIS patients [53]. These results corroborate our findings and suggest that NfL could be a robust biomarker for monitoring AIS prognosis at later timepoints and treatment effects and can be used as a benchmark for success in AIS drug development at the preclinical stage.

The 3-dose regimen of NSC EV was associated with reductions in serum NfL in the rat tMCAO model, indicative of reduced neuronal damage after stroke. NSC EV has been shown to reduce the extent of neural injury by suppressing inflammatory signaling cascades in the acute post-stroke period through the activity of therapeutic cargo (miRNAs and proteins) and surface molecules [5, 6]. Cell-based assays and animal stroke models conducted by our lab have elucidated several potential direct and indirect pathways by which NSC EV functions to preserve brain tissue and improve stroke outcomes [4–6]. NSC EV possess AMPase enzymatic function, which is involved in converting adenosine triphosphate (ATP) to anti-inflammatory adenosine, through the activity of CD73 on the EV membrane [54]. Furthermore, NSC EV reduces protein levels of proinflammatory C-C Motif Chemokine Ligand 2 (CCL2) in activated human microglia cells [55]. Reduced CCL2 signaling could lead to a more intact blood-brain barrier and less immune cell infiltration into the CNS, as well as reduced polarization of these immune cells to a pro-inflammatory phenotype [56, 57]. NSC EV mitigates the systemic immune response evident by significant reductions in pro-inflammatory circulating Th17 cells as well as significant increases in anti-inflammatory T regulatory cells and M2 macrophages in a rodent thromboembolic stroke model, leading to reduced infarct volume and preserved sensorimotor function [6]. Together, these mechanisms are thought to reduce infarct growth, and serum NfL levels may be a correlative biomarker associated with the reduced neuronal damage.

We found that all three NSC EV dosing regimens significantly reduced infarct volume at Day 3 post-stroke. This effect is notable given the timing of treatment initiation: whereas the 1-dose and 3-dose regimens began 4 h after reperfusion, the first dose of the 2-dose regimen was delayed until 24 h post-reperfusion. Although reduced infarct volume in the 2-dose group did not correlate with improvements in neurologic score or body weight, future studies will focus on optimizing functional assessments to confirm NSC EV–mediated effects when treatment is initiated at later time points. It is possible that more sensitive, or domain-specific, functional assessments such as the cylinder test or adhesive removal test could more robustly detect differences in the therapeutic effects of the three dose regimens. The ability of NSC EV to reduce infarct volume despite delaying the first administration to 24 h suggests potential utility in a broader patient population, including individuals who have missed the therapeutic window for tissue plasminogen activator [58].

There are limitations to this study. To minimize biological variability from sex hormones, we limited our cohort to male rats; future work should incorporate female models. This study could also be improved by correlating serum NfL levels with cerebrospinal fluid (CSF) NfL levels, as the latter is a more direct measure of axonal injury and the serum/CSF NfL ratio may be influenced by blood-brain barrier changes in the post-stroke sequelae. Furthermore, treatment groups were not randomized nor were investigators blinded. It is possible that the weak correlations for S100B and ICAM-1 may be due to insufficient power, increasing the risk of a Type II error. Finally, longitudinal studies assessing the durability of NfL as a biomarker in later stages post-stroke should also be conducted in the future.

In conclusion, we show for the first time in a rat stroke model that serum NfL is highly correlated to both infarct volume and body weight loss. Furthermore, we show that serum NfL performed as well as infarct volume in predicting a good outcome in stroked rats. These correlations were supported with evidence that NSC EV, a robust biologic therapy for AIS, significantly reduces serum NfL levels as well as preserves brain tissue and neurologic function. Overall, these data illustrate that serum NfL may be a useful biomarker to assess the therapeutic potential of investigational products for AIS to be directly translated to successful AIS clinical trials.

## Supplementary Information

Below is the link to the electronic supplementary material.

Supplementary Material 1

## Data Availability

Data generated from the current study is available from the corresponding author upon reasonable request.

## Abbreviations

AIS: acute ischemic stroke
ALS: amyotrophic lateral sclerosis
ANOVA: analysis of variance
ATP: adenosine triphosphate
AUC: area under curve
CCA: common carotid artery
CCL2: C-C motif chemokine ligand 2
ECA: external carotid artery
EV: extracellular vesicle
EVT: endovascular thrombectomy
HLVe: hemispheric lesion volume with edema correction
ICA: internal carotid artery
ICAM-1: intercellular adhesion molecule 1
IFNƴ: interferon-gamma
IL-1β: interleukin-1 beta
IL-6: interleukin-6
MCA: middle cerebral artery
mRS: modified rankin scale
NfL: neurofilament light chain
NIH: National Institutes of Health
NIHSS: NIH stroke scale
NSC: neural stem cell
ROC: receiver operating characteristics
S100B: S100 calcium binding protein
SEM: standard error of the mean
TBI: traumatic brain injury
TFF: tangential flow filtration
tMCAO: transient middle cerebral artery occlusion
TNFα: tumor necrosis factor-alpha
TTC: triphenyl tetrazolium chloride
