# Supplementary material for: Serum Neurofilament Light Chain Predicts Stroke Outcome and is a Potential Marker for Treatment Effects of Neural Stem Cell-derived Extracellular Vesicles in a Rat Stroke Model
Source: Transl Stroke Res. 2026 May 8;17(3):54. doi: 10.1007/s12975-026-01445-6 (PMC13156156; doi:10.1007/s12975-026-01445-6)
Supplement: Supplementary file 1 — Supplementary Material 1 [file 12975_2026_1445_MOESM1_ESM.docx]

**Supplementary Figures**

Supplementary Figure 1


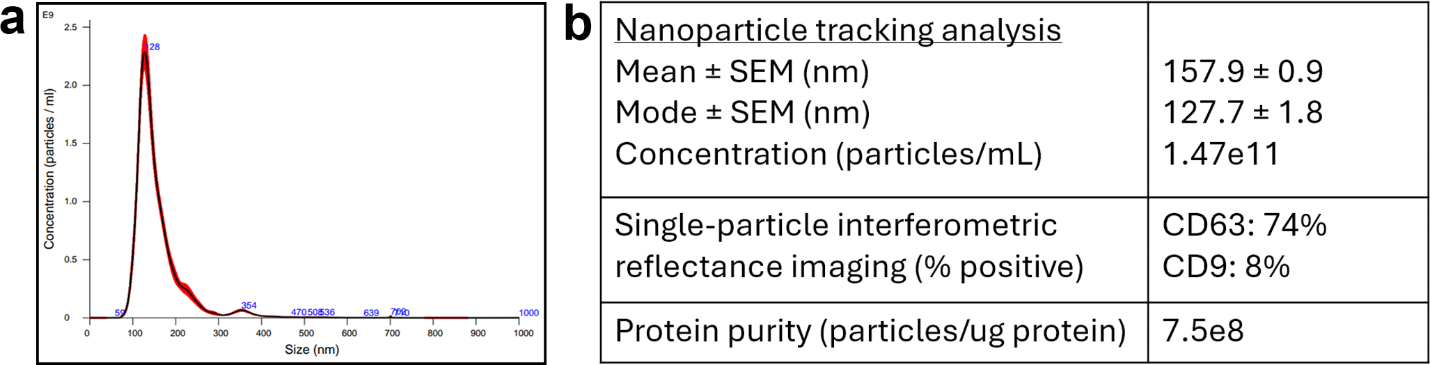


Supplementary Figure 2


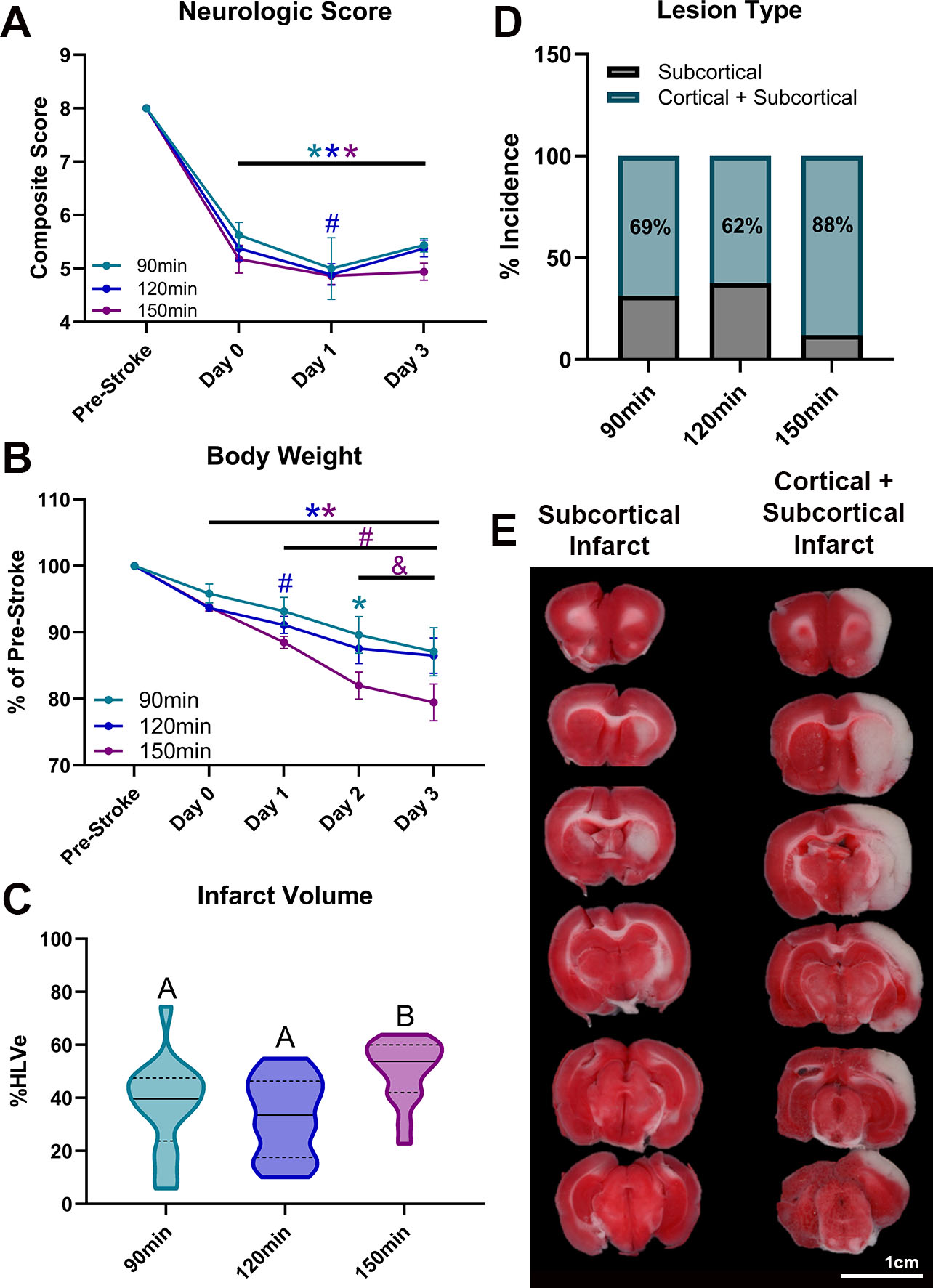


Supplementary Figure 3


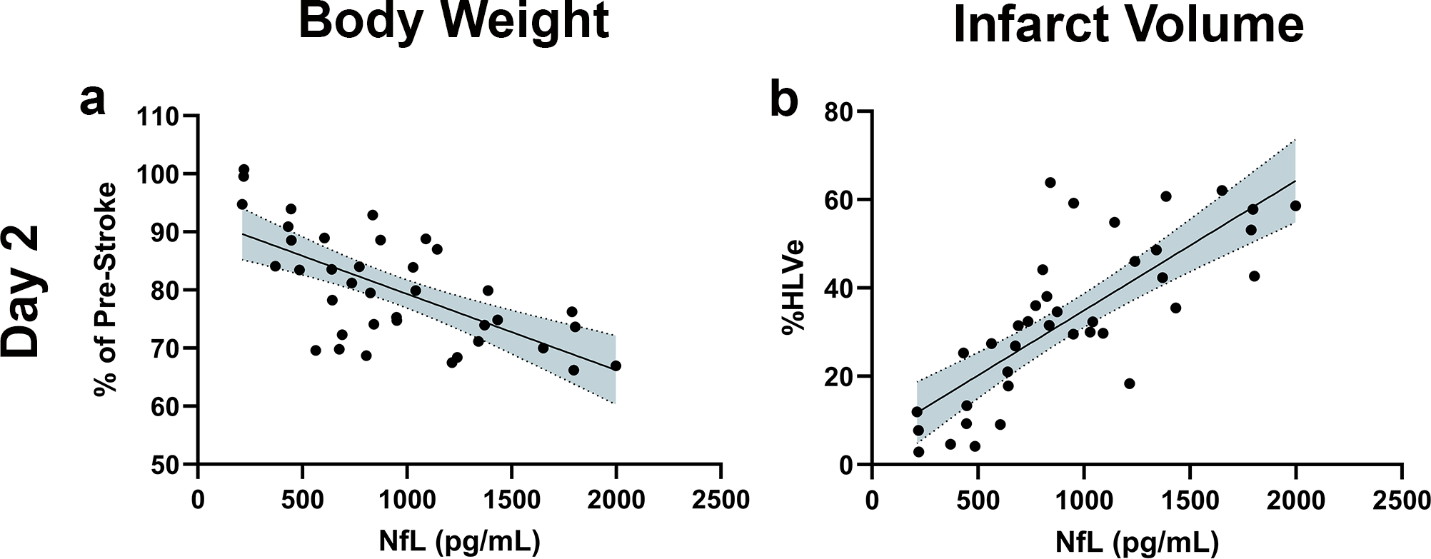


Supplementary Figure 4


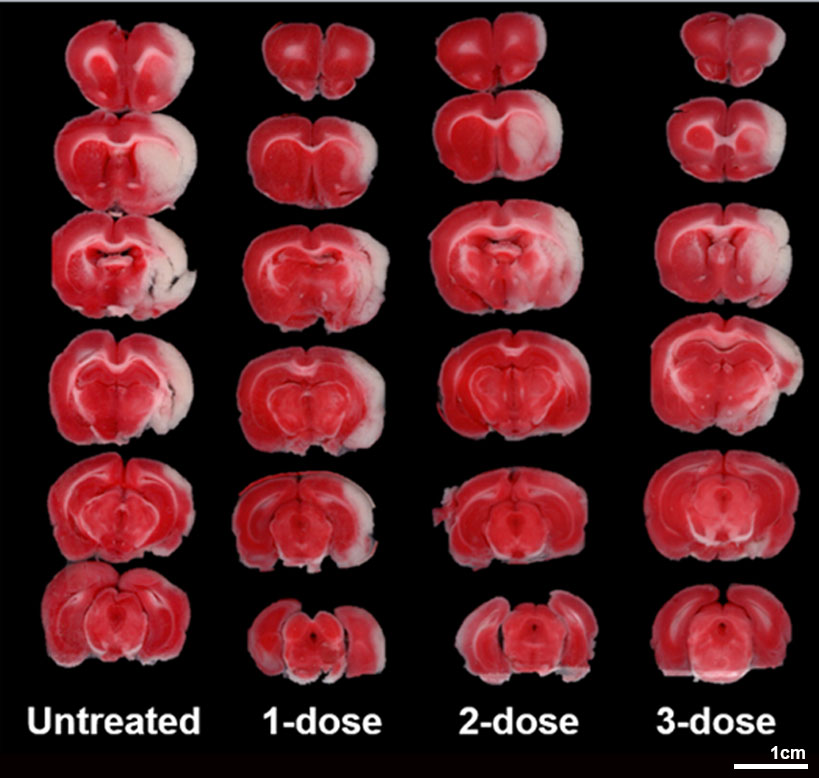


**Supp. Fig. 1** (**a**) NSC EV characterization nanoparticle tracking analysis and (**b**) size, concentration, protein marker and purity specifications.

**Supp Fig. 2** Increasing occlusion time to 150-minutes results in higher stroke-induced weight loss and infarct volume, as well as decreased incidence of subcortical infarcts. (**a**) Experimental outline showing pharmacological time points relative to tMCAO surgery. (**b**) Neurologic score collected pre-stroke and Day 0, 1, and 3. (**c**) Body weight data collected pre-stroke and Day 0, 1, 2, and 3. (**d**) Quantification of cerebral infarct volume on Day 3. (**e**) Occurrence of subcortical vs. cortical + subcortical lesion types for all occlusion times. (**f**) Representative images of TTC-stained coronal brain slices of subcortical only infarct (**left**) and infarct that extends to cortical and subcortical regions (**right**). *n*=16 for the 90-minute group; *n*=16 for the 120-minute group; *n*=17 for the 150-minute group. Data for **b**, **c**, and **d** expressed as Mean ± SEM; Data for **e** expressed as a percentage of cortical + subcortical strokes. * indicates *p* < 0.05 compared to pre-stroke; # indicates *p* < 0.05 compared to Day 0; & indicates *p* < 0.05 compared to Day 1. For panel **a**, ***BW:*** body weight, ***NS:*** neurologic score, and ***BC:*** blood collection. For panel **d**, levels not connected by the same letter are significantly (*p* < 0.05) different.

**Supp Fig. 3** Day 2 serum NfL is correlated with body weight and infarct volume. Correlation of Day 2 NfL with (**a**) body weight or (**b**) infarct volume (*n*=38).

**Supp Fig. 4** Representative images of coronally sectioned, TTC-stained brain slices for an untreated animal and animals treated with 1-, 2-, or 3-doses of NSC EV.
